# Evaluating Palliative Care Needs in Patients with Advanced Non-Malignant Chronic Conditions: An Umbrella Review of Needs Assessment Tools

**DOI:** 10.3390/healthcare14010046

**Published:** 2025-12-24

**Authors:** Chrysovalantis Karagkounis, Stephen Connor, Danai Papadatou, Thalia Bellali

**Affiliations:** 1 Nursing Department, International Hellenic University, Sindos, 57400 Thessaloniki, Greece; thalia@ihu.gr; 2 Worldwide Hospice Palliative Care Alliance (WHPCA), 34-44 Britannia Street, London WC1X9JG, UK; sconnor@thewhpca.org; 3 Nursing Department, National and Kapodistrian University of Athens, Goudi, 11527 Athens, Greece; dpap@nurs.uoa.gr; 4 Department of Health Sciences, European University Cyprus, Engomi, 1516 Nicosia, Cyprus

**Keywords:** assessment, identification of palliative care needs, long-term illnesses, patients with advanced non-malignant chronic conditions, needs assessment tools

## Abstract

**Highlights:**

**What are the main findings?**

- This umbrella review identified 35 needs assessment tools for patients with advanced non-malignant chronic conditions, with only a limited number integrating both general and disease-specific palliative care indicators.
- Significant gaps were found in the psychometric evidence of most tools, with NAT: PD-HF, SPICT, and NECPAL emerging as the most promising for this patient population.

**What are the implications of the main findings?**

- Early identification of palliative care needs should prioritize tools that predict functional decline and incorporate both general and disease-specific indicators to support timely and targeted interventions.
- Further research is required to strengthen the psychometric properties and clinical utility of existing tools and to develop more holistic assessment tools covering physical, psychological, social, and spiritual domains.

**Abstract:**

Background/Objectives: Patients with advanced non-malignant chronic conditions experience illness burdens and palliative care needs comparable to those of oncology patients, yet palliative care is often introduced late. Identifying individuals with potential palliative care needs is complex, and although multiple tools exist, the most appropriate approach for assessing needs in this population remains unclear. This umbrella review aimed to identify and evaluate tools used to systematically assess palliative care in adults with advanced non-malignant chronic conditions, with a specific focus on their content, structure, and psychometric properties. Methods: An umbrella review of systematic reviews was conducted in accordance with the Joanna Briggs Institute (JBI) guidance. Four electronic databases (Cochrane Library, CINAHL, PubMed, and PsycINFO) were searched from inception to 30 June 2025. Eligible systematic reviews were screened, critically appraised, and synthesized narratively. Results: Seven systematic reviews met the inclusion criteria, collectively identifying 35 unique needs-assessment tools. Five tools (SPICT, GSF-PIG, QUICK GUIDE, NECPAL, and P-CaRes) incorporated both general and disease-specific palliative care indicators. At the same time, four (PC-NAT, SPEED, NAT, and IPOS) addressed needs across physical, psychological, social, and spiritual domains. Psychometric data were available for six tools across three reviews. The original NAT and SPICT demonstrated good reliability; however, the Dutch version of the NAT showed poor validity. SPEED and one unnamed palliative care tool showed good reliability, whereas the Surprise Question demonstrated unclear validity. Italian-SPICT and Israeli-NECPAL exhibited strong content validity. Conclusions: Despite limited evidence, the NAT: PD-HF shows particular promise for identifying palliative care needs in patients with heart failure. Tools such as SPICT and NECPAL are widely used and adapted for advanced non-malignant chronic conditions, but further psychometric evaluation is required. Additional studies are needed to clarify the clinical utility of these tools for broader implementation in assessing palliative care needs.

## 1. Introduction

Chronic conditions are of increasing concern as the global population ages [1]. Conditions such as heart failure, chronic obstructive pulmonary disease, and dementia are prevalent and associated with substantial symptom burden, high utilization of healthcare services, and reduced quality of life [2,3]. Palliative care aims to enhance quality of life, alleviate suffering, and support decision-making for individuals with serious illness and their caregivers [4]. While palliative care research has traditionally focused on oncology, the population living with advanced non-malignant conditions is substantially larger and experiences comparable needs. Applying evidence generated primarily from malignant diseases can be misleading, given the unique, fluctuating trajectories and functional decline patterns observed in non-malignant conditions [5,6,7,8]. Patients with advanced non-malignant chronic conditions frequently experience heightened physical and psychological symptom burden [9,10,11,12]. Τhere is growing evidence that timely palliative care involvement improves quality of life, reduces unnecessary hospitalizations, and may even prolong survival [13,14,15,16,17,18,19]. Despite this, palliative care for non-malignant diseases is often initiated late, inconsistently, and inequitably—a pattern pointing to systemic challenges in early identification [20].

In this umbrella review, the term palliative care needs refers to the constellation of physical symptoms (e.g., pain, breathlessness), psychological distress (e.g., anxiety, depression), social support needs, and existential or spiritual concerns [4]. These needs are often multidimensional, persistent, and episodic, arising throughout the course of progressive chronic illness [12,21,22]. Their early recognition is essential for integrated, person-centred care [10]. Generalist palliative care—delivered by primary care clinicians, nurses, and other non-specialists—plays a key role in this process, while specialist services are typically reserved for complex or refractory needs [5,6,23].

A key barrier to delivering timely palliative care is the systematic identification of patients who may benefit from it [24,25]. Not all individuals with advanced non-malignant conditions require palliative care at a given moment, and busy clinical environments make comprehensive assessments impractical for all patients [26]. This underscores the need for efficient, evidence-based tools that support clinicians in proactively and consistently recognizing potential palliative care needs. A growing array of assessment tools has been developed for this purpose, varying widely in scope, structure, and intended use. Some tools focus on illness severity, disease progression, or frailty; others are tailored for specific clinical settings (e.g., emergency department, intensive care) [27] or particular patient groups, such as those with interstitial lung disease [28] or older adults [29]. Some tools are clinician-administered, whereas others can be self-reported by patients or caregivers [30,31]. Importantly, only a minority have been systematically validated for non-malignant conditions, and many remain underused in clinical practice. Existing systematic reviews tend to examine isolated conditions or narrow subsets of tools [32,33].

Given the multiplicity of tools, their conceptual differences, and the lack of consolidated evidence on their suitability for advanced non-malignant chronic conditions, a comprehensive synthesis is needed. Understanding which tools support early identification, which provide multidimensional assessment, and which combine both functions is crucial for guiding clinical practice and future research. Therefore, this umbrella review aimed to systematically identify and evaluate tools used to assess palliative care needs in adults with advanced non-malignant chronic conditions, with particular attention to their content, structure, purpose, target population, and psychometric properties. Specifically, this review sought to answer the following research question:

What tools are available for assessing palliative care needs in patients with advanced non-malignant chronic conditions, and what evidence exists regarding their content, structure, purpose, and psychometric properties?

## 2. Materials and Methods

### 2.1. Design

An umbrella review is a comprehensive examination of evidence compiled from multiple systematic research syntheses focusing on different interventions or perspectives on a particular topic [34]. Such reviews emphasize clinical conditions that are broad enough to have been evaluated with multiple interventions and aim to synthesize this diverse evidence base into a single accessible summary [34]. In the context of palliative care needs assessment—where numerous systematic reviews have examined different tools, populations, and settings using heterogeneous methods and sometimes reporting conflicting findings—an umbrella review provides a higher-level, methodologically rigorous synthesis capable of integrating and comparing this fragmented evidence base [34,35,36].

Accordingly, in this study, we applied an umbrella review design to collate and synthesize findings from existing systematic reviews on tools used to identify palliative care needs in adults with advanced non-malignant chronic conditions. This approach enables a broad overview of the available tools, their use across different care settings, and their psychometric properties [34], while allowing healthcare professionals to efficiently survey published evidence relevant to the research problem and identify the most suitable approaches for clinical decision-making [35].

The review process followed the Joanna Briggs Institute (JBI) methodological guidance for umbrella reviews [34,37]. This review was prospectively registered in PROSPERO (CRD42024553053) and conducted according to the PRIOR (Preferred Reporting Items for Overviews of Reviews) statement to ensure methodological transparency in reporting objectives, methods, results, and conclusions. Critical methodological components—including eligibility criteria based on PICOTSS parameters, the search strategy, study screening and selection procedures, data extraction, risk of bias assessment, and the synthesis plan—were predefined in the registered protocol. The search and reporting procedures also complied with PRISMA 2020, with particular attention paid to documenting the search process and study flow to enhance clarity and rigor [38].

### 2.2. Search Strategy

The umbrella review search strategy was designed according to JBI’s methodological guidelines, and its conduct adhered to the standards outlined in the PROSPERO-registered protocol. Given our aim to evaluate needs-assessment tools that reflect current clinical practice, eligibility was restricted to systematic reviews published between 2014 and 2024. Older tools were excluded because they are frequently outdated, insufficiently validated, or no longer aligned with contemporary palliative care models. Search reporting followed the PRISMA 2020 guidelines to ensure transparency and reproducibility [38]. The search was conducted by two independent reviewers across all selected databases using systematic combinations of a priori-defined keywords, Boolean operators, and eligibility criteria. Both the inclusion and exclusion criteria were guided by the PICOTSS framework, which refers to Population, Intervention (or Indicator), Comparison, Outcome, Time, Setting, and Study Design (Table 1). Restricting inclusion to tools evaluated in systematic reviews was intentional to preserve methodological rigor; however, we acknowledge that this decision may have led to the exclusion of newly developed tools that have not yet undergone systematic review.

We systematically reviewed four databases—the Cochrane Library (Table 2), CINAHL (Table S2), PubMed (Table S3), and PsycINFO (Table S4)—for studies meeting the eligibility criteria (Table 1). The database search was conducted from inception to 30 June 2025 to ensure that all potentially relevant reviews were captured. However, eligibility for inclusion was restricted a priori (as specified in the PROSPERO protocol) to systematic reviews published within the last ten full calendar years (2014–2024), to reflect contemporary clinical practice and avoid distortions introduced by partial publication years. One exception was made for a single earlier review published in 2013, which was retained because it represents the only systematic review covering several core palliative care identification tools (e.g., NECPAL, SPICT, RADPAC) for which no subsequent or updated systematic reviews exist. Excluding this review would have resulted in the omission of key tools still in current international use and would have introduced a content-related bias. This rationale is consistent with JBI, which notes that limits on a search strategy—such as the use of date restrictions—should be appropriate and explicitly justified, as guidance that allows the inclusion of older reviews when they provide unique, irreplaceable evidence [37].

### 2.3. Study Selection and Quality Appraisal

Two members of the review team independently conducted a critical appraisal of the reviews, utilizing a modified version of the JBI Critical Appraisal Tool for Systematic Reviews [37]. The tool incorporates specific questions designed to verify the use of appropriate search methods and a systematic approach to reviewing data before extraction and synthesis. When discrepancies arose in the appraisal outcomes, they were resolved through discussion; if necessary, two additional reviewers were consulted.

### 2.4. Data Extraction and Synthesis

The characteristics of the included reviews and the needs assessment tools were collected before synthesis. Whenever possible, information on psychometric properties (e.g., validity, reliability, sensitivity, specificity) was also extracted; however, such data were not consistently reported across all reviews, and their presence or absence was explicitly noted in the synthesis. One reviewer extracted data on review characteristics, tool features, target populations, and reported psychometric properties, and three additional reviewers independently checked the extracted dataset for accuracy and completeness. The verification process addressed only the correctness and completeness of the extracted data, not the quality or suitability of the individual tools; any discrepancies were resolved by consensus.

Due to the heterogeneity of the included reviews in terms of populations, settings, and outcomes, and the predominantly qualitative nature of the reported findings, we used a narrative synthesis approach. We organized the tools in a comparative matrix according to four key domains: (a) scope (general vs. disease-specific); (b) purpose (early identification vs. comprehensive multidimensional needs assessment vs. end-of-life planning); (c) target population (e.g., heart failure, COPD, dementia); and (d) reported psychometric properties. When available, we extracted summary psychometric indices (e.g., Cronbach’s alpha, inter-rater reliability, and validity types) directly from the systematic reviews. 

Given that our unit of analysis was systematic reviews rather than primary psychometric or diagnostic accuracy studies, structured tools such as the COSMIN Risk of Bias checklist and QUADAS-2 were not applied; instead, we relied on the psychometric information as summarized by the included reviews. The narrative synthesis was conducted independently by two reviewers, with any disagreements resolved through discussion with a third reviewer. In line with our overarching aim, we did not conduct new psychometric analyses but instead integrated and interpreted the available evidence on validity and reliability reported in the systematic reviews. To enhance comparability and practical utility, the identified tools were ultimately grouped by target population, tool type (general vs. disease-specific), and primary purpose (early identification, comprehensive multidomain assessment, or mixed purpose).

## 3. Results

### 3.1. Selected Studies

**Figure 1:**
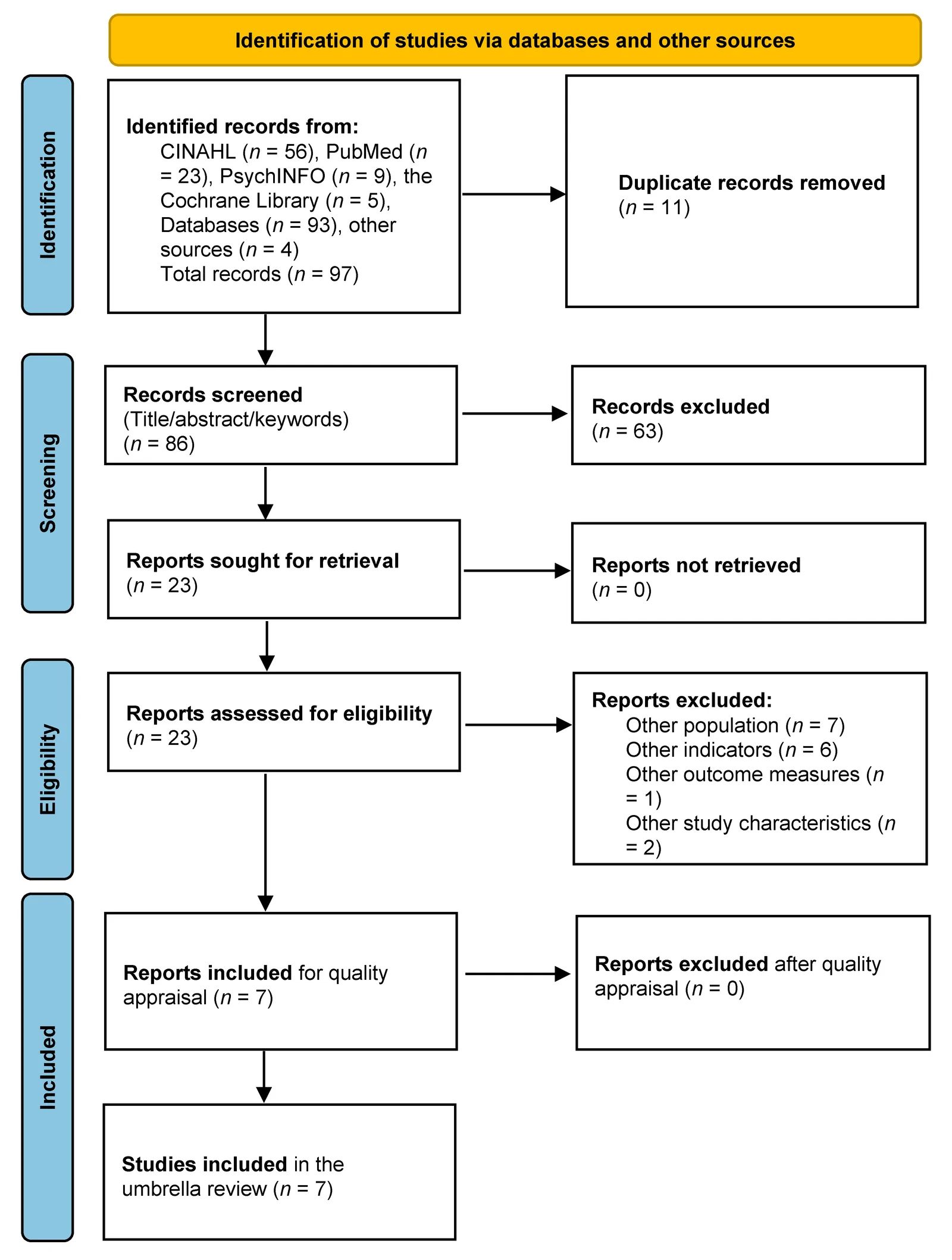
PRISMA flowchart outlining the selection process, critical appraisal, and data extraction.

The search strategy identified 97 records across the four databases and additional sources. After removing 11 duplicates, 86 records were screened based on title, abstract, and keywords. Twenty-three full-text articles were assessed for eligibility, of which 16 did not meet the inclusion criteria. The remaining seven systematic reviews were critically appraised and included in the umbrella review (Figure 1, PRISMA flowchart).

### 3.2. Characteristics of the Included Studies

Seven reviews were included: three systematic literature reviews, two systematic reviews with narrative synthesis, one systematic review with meta-analysis, and one mixed-method systematic review (Table 3). Most were published between 2019 and 2023, with one older review from 2013. Five reviews appeared in palliative care journals (e.g., BMJ Supportive & Palliative Care, Palliative Medicine, BMC Palliative Care), and two in broader medical journals (Heart Failure Reviews, Academic Emergency Medicine). Most reviews searched four to seven databases, and all included PubMed/MEDLINE and Embase in their strategies. Three reviews reported psychometric data for at least one tool, while all seven reported on multiple needs-assessment tools.

### 3.3. Risk of Bias Across Studies

The risk of bias for the included reviews is summarized in Table S5. All seven systematic reviews met at least 9 of the 11 JBI critical appraisal criteria, indicating overall good methodological quality. Methodological limitations were most often related to the absence of an explicitly formulated research question or eligibility criteria structured in PICOTSS format, as well as incomplete reporting of publication bias assessment in several reviews (Table S5, Q1, Q9) [44]. In one review, the procedures used to minimize data extraction errors were insufficiently detailed, and publication bias assessment was not reported (Q7, Q9) [32]. Across several reviews [32,39,42,43,44], research questions were not fully specified in PICOTSS format, although study objectives were clearly articulated. Only one review presented uncertainty regarding whether specific directives for future research were appropriately addressed (Q11) [44]. Overall, the methodological quality of the included reviews was judged to be sufficient to support synthesis of findings on needs-assessment tools for adults with advanced non-malignant chronic conditions, given that all reviews demonstrated generally robust methodological standards and captured a diverse range of tools across multiple healthcare settings.

### 3.4. Characteristics of the Needs Assessment Tools

A total of 35 needs-assessment tools were identified across North America, Europe, Asia, and South America, developed for use in diverse chronic non-malignant and mixed populations. Most tools were originally developed in English, with many translated into multiple languages, supporting international applicability. Tools varied in format—predominantly paper-based, with a smaller number of electronic versions—and were designed for use in a range of settings including primary care, hospitals, emergency departments, hospice, and community services. Detailed characteristics for all tools are presented in Table 4.

To facilitate comparison, tools were classified according to target population (e.g., heart failure–specific, dementia-specific, frailty, intellectual disability), scope (general vs. disease-specific; mixed where both were combined), and primary purpose. Purpose was categorized as: (i) early-identification tools designed to flag patients who may benefit from a palliative approach; (ii) comprehensive multidomain assessment tools that systematically evaluate physical, psychological, social, and spiritual needs; and (iii) mixed-purpose tools, including tools assessing functional status, frailty, or quality of life, or tools combining screening with partial needs assessment. Some tools served more than one function. A summary classification by purpose is provided in Table 5.

Across the 35 tools, most were general in scope, while a smaller number were disease-specific or combined general and disease-specific indicators. Early-identification and end-of-life–focused tools constituted a substantial proportion of tools, whereas comprehensive multidomain assessments represented a smaller subset. Several tools combined early-identification with broader assessments, although no single tool explicitly addressed all three purposes concurrently. Paper-based formats predominated, with relatively few electronic versions and limited reporting of mixed formats. Most tools were clinician-administered, and only a minority permitted completion by patients or caregivers. Only a small subset incorporated a structured follow-up or action-planning component, indicating a persistent implementation gap between needs identification and care delivery.

Several tools included prognostic triggers such as the SQ, and when completion time was reported, some tools could be completed within minutes, supporting feasibility in routine clinical practice. While some tools combined general and disease-specific indicators, others relied exclusively on markers of decline or functional deterioration. Only a limited number of tools assessed all four core palliative care domains. Approximately two-thirds of the tools applied explicit criteria or cut-off values for identifying potential palliative care needs, whereas others provided structured clinical profiles without specified thresholds (Table 4).

### 3.5. Psychometric Properties of the Needs Assessment Tools

Overall, psychometric evidence for the identified tools was limited and unevenly reported across the included reviews (Table 6). Only three of the seven reviews provided data on validity or reliability, allowing tools to be grouped into three categories: (i) tools with the strongest and most consistent psychometric support, (ii) tools with weak or inconsistent evidence, and (iii) tools for which no psychometric evaluation was reported at the review level.

#### 3.5.1. Tools with Strongest Validity and/or Reliability

Evidence of good psychometric performance was reported for several tools. The original NAT: PD-HF demonstrated good reliability and validity across all evaluated domains [41]. The 13-item SPEED tool and the Peruvian Palliative Care Tool (Unnamed) also showed good reliability [42]. Xie et al. [44] reported good reliability for the original SPICT and very good content validity for the Italian-SPICT and Israeli-NECPAL, indicating solid measurement properties for these tools.

#### 3.5.2. Tools with Inconsistent or Weak Psychometric Findings

Some tools showed mixed psychometric profiles. The Dutch version of NAT: PD-HF demonstrated poor validity despite the strong performance of the original version [41]. The SQ, although widely used, showed doubtful construct validity as a standalone prognostic measure, with weak correlations against several comparator tools [42].

#### 3.5.3. Tools Lacking Psychometric Evaluation in the Included Reviews

For the remaining tools, including widely used tools such as IPOS, ESAS, PPS, GSF-PIG, NECPAL (original version), RADPAC, and many others, the included systematic reviews did not report psychometric findings. This absence reflects limitations in reporting within the reviews rather than an absence of validation studies in the broader literature. Table 6 summarizes the available psychometric evidence extracted exclusively from the systematic reviews included in this umbrella review.

It should be noted that widely used tools such as IPOS and ESAS have established psychometric support in primary validation studies; however, this evidence was not consistently reported in the included systematic reviews. Because our umbrella review relied exclusively on data extracted from systematic reviews, gaps in Table 5 and Table 6 mainly reflect limitations in reporting at the review level rather than the absence of underlying validation work. Future syntheses that integrate both umbrella review data and primary psychometric studies would be valuable for generating firm tool-specific recommendations.

## 4. Discussion

This umbrella review synthesizes evidence from seven systematic reviews and maps 35 needs-assessment tools designed to identify potential palliative care needs among adults with advanced non-malignant chronic conditions. By integrating findings across heterogeneous reviews—each differing in scope, definitions, populations, and methodological emphasis—this review offers a level of conceptual consolidation not previously available in the literature. Whereas individual reviews have focused narrowly on specific clinical groups (e.g., frailty, heart failure, emergency care) or on isolated types of tools, the present synthesis brings together the full spectrum of tools spanning early identification, multidimensional assessment, and end-of-life planning. In doing so, it highlights cross-cutting limitations, persistent operational barriers, and long-standing conceptual inconsistencies in how palliative care needs are defined and assessed.

A central insight emerging from this synthesis is that palliative care needs in non-malignant conditions cannot be reliably identified using mortality prediction alone. Patients with advanced heart failure, COPD, neurodegenerative disorders, frailty, and multimorbidity experience highly variable and non-linear trajectories, with fluctuating needs that often diverge from classical terminal decline patterns observed in cancer [23]. Reliance on mortality forecasting or deterioration prediction therefore reflects a conceptual lag, given that anticipatory, needs-based assessment—not survival estimation—is the essence of contemporary palliative care frameworks [40,77]. Tools combining both general and disease-specific indicators (e.g., SPICT, GSF-PIG, QUICK GUIDE, NECPAL, P-CaRES) show stronger conceptual alignment with these fluctuating patterns; however, they represent only a minority of the tools identified.

The widespread incorporation of the SQ across nearly one-third of included tools underscores its appeal as a rapid screening mechanism, especially in time-pressured environments such as emergency departments [42]. Yet evidence consistently demonstrates substantial limitations. SQ’s predictive accuracy varies markedly across malignant and non-malignant trajectories, with poorer performance in chronic non-cancer conditions [26]. Its utility is further constrained by heavy reliance on clinician intuition, which is influenced by experience, familiarity with specific disease trajectories, and comfort with prognostication [26]. The result is wide variability and a risk of both false-positive and false-negative identification, raising questions about its suitability as a standalone trigger. Tools integrating the SQ within a broader clinical framework (e.g., P-CaRES, Rainone tool) partially mitigate these limitations by contextualizing prognostic intuition with objective indicators.

Operational characteristics of the tools further illuminate challenges in clinical adoption. Completion times range from less than two minutes (ESAS, CSHA-CFS) to approximately 30 min for culturally adapted versions of NAT: PD-HF [21]. These discrepancies reflect a fundamental tension between feasibility and comprehensiveness. Tools assessing multidimensional needs require higher cognitive load, patient involvement, and clinical interpretation, and may be difficult to integrate into routine workflows without dedicated resources or multidisciplinary structures [33,78]. Only a minority of tools include an explicit action-planning component (e.g., NAT: PD-HF, SPEED), despite evidence that such mechanisms enhance clinical integration and care coordination [79]. In contrast, tools such as IPOS—despite well-established psychometric properties—lack formally embedded follow-up mechanisms, which may limit their practical impact in busy clinical environments [23].

A notable conceptual limitation across the identified tools is the incomplete incorporation of holistic palliative care domains. Only four tools (PC-NAT, SPEED, NAT: PD-HF, IPOS) explicitly cover physical, psychological, social, and spiritual dimensions. This reflects a broader systemic challenge in operationalizing the biopsychosocial-spiritual model in routine care [22,80]. Psychosocial and spiritual needs often co-occur with physical symptom burden in patients with advanced non-malignant conditions [81,82,83,84,85] yet remain under-represented in most tools. Failure to capture these domains risks delayed referral, fragmented assessment, and suboptimal symptom management [86,87]. The omission of spiritual and social dimensions in particular points to a disconnect between theoretical palliative care models and the practical constraints of clinical assessment. 

Psychometric evaluation remains another area of concern. Despite decades of tool development, rigorous validation evidence is scarce. The strongest psychometric support identified in the included reviews relates to the original NAT: PD-HF, SPEED, the Peruvian Palliative Care Tool, SPICT, Italian-SPICT, and Israeli-NECPAL [41,42,44]. Weak or inconsistent findings, such as the poor validity of the Dutch NAT: PD-HF, illustrate the consequences of insufficient cultural adaptation, inadequate sample sizes, and limited methodological rigor [21,41]. The absence of psychometric reporting for widely used tools such as IPOS, ESAS, PPS, GSF-PIG, and NECPAL (original version) in the included reviews reflects methodological constraints in systematic review practices rather than an absence of validation work in primary literature. Nonetheless, these persistent gaps signal a structural misalignment between tool development, validation, and implementation.

Finally, this umbrella review reveals significant conceptual inconsistency in how “palliative care needs” are defined across the literature. Some reviews operationalize needs narrowly in terms of proximity to end-of-life [39], others emphasize early identification [44], while others link needs primarily to prognostic triggers [24,25]. Such variation mirrors ongoing debate in the field regarding the scope and timing of palliative care, especially beyond oncology [88]. The structured classification used in this review (by purpose, scope, and population) offers a more coherent framework through which tools can be meaningfully compared and selected for clinical use.

### 4.1. Implications

The findings of this umbrella review have several implications for clinical practice, tool development, health systems, and future research. In clinical practice, the use of tools that incorporate both general and disease-specific indicators and that assess needs across physical, psychological, social, and spiritual domains should be prioritized, as these tools offer a more holistic and clinically meaningful understanding of patient needs. Reliance on prognostic triggers such as the SQ should be supplemented with broader assessment approaches, particularly for non-malignant chronic conditions where disease trajectories are highly variable and prognostication remains uncertain. Tools that include explicit mechanisms for documenting or triggering action plans may further support decision-making and care coordination by linking identified needs to concrete clinical responses.

From a tool development perspective, future tools would benefit from being firmly grounded in a biopsychosocial-spiritual framework to ensure comprehensive coverage of the full spectrum of patient needs. Embedding structured action pathways within these tools is likely to enhance their clinical utility, while rigorous psychometric testing—including validity, reliability, and cultural adaptation—should be integral to their development to ensure their applicability across diverse populations and settings. At the health system and policy level, effective integration of validated needs-assessment tools into routine care requires adequate training, resource allocation, and supportive digital infrastructures. Policies that promote early identification and multidimensional assessment may reduce variability in care by decreasing reliance on clinician intuition alone. Achieving consistency in the use of such tools also depends on the standardization of terminology related to “palliative care needs,” which is currently used inconsistently across the literature and clinical practice.

Finally, several research priorities emerge. There is a clear need for high-quality validation studies, especially for tools that are widely implemented but have limited psychometric evaluation in existing systematic reviews. Future research should compare tools in real-world settings to determine their feasibility, performance, and impact on patient outcomes. Studies exploring how multidimensional needs assessment can be operationalized within time-constrained clinical environments will be essential for improving applicability. Moreover, longitudinal investigations are required to understand how these tools perform across the fluctuating trajectories typical of advanced non-malignant chronic conditions.

### 4.2. Strengths and Limitations

To our knowledge, this is the first umbrella review to systematically synthesize tools designed to identify potential palliative care needs in adults with advanced non-malignant chronic conditions. A comprehensive search strategy was employed across four major databases (Cochrane Library, CINAHL, PubMed, PsycINFO), and the quality of the included systematic reviews was independently appraised by multiple reviewers in accordance with JBI and PRISMA guidance. In contrast to the quality appraisal process for the systematic reviews, the assessment of “validation studies” within this umbrella review refers to primary research reporting psychometric properties (e.g., reliability, validity), which is essential for determining the appropriateness and methodological strength of needs-assessment tools in clinical practice.

Despite these strengths, several limitations should be acknowledged. First, although every effort was made to retrieve all relevant systematic reviews, some omissions are possible. Because the umbrella-review design relies exclusively on published systematic reviews, emerging tools such as ID-Pall [89] or newly validated tools such as IPOS [90] were not captured unless already included within an eligible review. This introduces a structural limitation, whereby innovative or updated tools may be underrepresented due to the inherent lag between primary validation work and inclusion in systematic reviews.

Second, the depth and completeness of psychometric data available in this umbrella review were constrained by the reporting practices of the included systematic reviews. Only three of the seven reviews provided detailed psychometric information, limiting the extent to which meaningful comparisons could be drawn across tools. Although all available psychometric data were extracted and summarized (Table 6), the absence of standardized reporting (e.g., Cronbach’s alpha, inter-rater reliability, criterion validity) in several reviews reduced comparability. Moreover, three of the included systematic reviews were based on searches older than five years; they were retained because they contributed unique information about widely used tools still in practice, though future umbrella reviews may benefit from more targeted updates.

Third, several methodological considerations apply to the search strategy. By restricting inclusion to studies published in English, the review may be subject to language bias. Relevant studies published in other languages—especially given the global development of palliative care tools—may therefore have been excluded. We also excluded full-text-unavailable studies, which, while ensuring rigorous data extraction, may have inadvertently omitted relevant evidence. Additionally, our keyword strategy may not have fully captured semantic variations in terminology (e.g., “screening tools,” “palliative needs,” “supportive care”), leaving open the possibility of missing studies that use alternate descriptors. Future reviews may mitigate these issues through broader terminology mapping and inclusion of grey literature.

Fourth, this umbrella review did not evaluate clinical performance metrics (sensitivity, specificity, PPV, NPV, accuracy) because these were rarely reported in the systematic reviews and were not the primary focus of the synthesis. Nevertheless, these measures are critical for determining the practical applicability of screening tools, and targeted searches of primary literature may be required to supplement future analyses. Another limitation concerns the potential for duplication bias, whereby the same primary studies may have been included across multiple systematic reviews. Such overlap, which is inherent in umbrella reviews, may disproportionately influence the representation of evidence for certain tools. Although we mitigated this by synthesizing at the level of the systematic review rather than weighting individual primary studies, some degree of duplication remains possible.

Finally, an overarching challenge arose from the conceptual heterogeneity in how “palliative care needs” were defined across the systematic reviews—from end-of-life identification [39] to early needs screening [44]. Although we addressed this issue by categorizing tools according to scope, population, and purpose, differences in underlying theoretical models and operational definitions may still influence interpretation. The limited and inconsistent reporting of psychometric properties across tools further restricts the ability to formulate strong, generalizable recommendations for clinical implementation.

## 5. Conclusions

This umbrella review synthesized evidence from seven systematic reviews and identified thirty-five tools developed or used to detect potential palliative care needs in adults with advanced non-malignant chronic conditions. Although these tools represent substantial effort to standardize early identification, most lack robust psychometric evaluation, limiting confidence in their accuracy and consistency. Among the available tools, the original NAT: PD-HF demonstrates the strongest evidence base for heart failure populations, while SPICT and NECPAL—particularly their validated international adaptations—appear most suitable for broader non-malignant chronic conditions due to their integration of general and disease-specific indicators. For clinical practice, the findings highlight the need to favour tools that offer multidimensional assessment, incorporate both general and condition-specific indicators, and provide actionable outputs that support care planning. Clinicians should avoid relying solely on prognostic triggers such as the SQ and instead adopt tools that enable systematic, holistic identification of evolving needs across physical, psychological, social, and spiritual domains. At the same time, the field requires more rigorous validation and comparative effectiveness research. Future work should evaluate tools within a shared conceptual and clinical framework, incorporating psychometric testing, diagnostic accuracy, and real-world usability across diverse settings. Strengthening this evidence base will allow clinicians and health systems to select tools that are not only conceptually sound but also operationally feasible and capable of improving patient outcomes.

## Figures and Tables

**Table 1:** Selection criteria for inclusion or exclusion of reviews.

	Inclusion Criteria	Exclusion Criteria
Population	Adults (18 years and older) diagnosed with advanced non-malignant chronic conditions potentially associated with palliative care needs	Birth–5 years, Children (6–12 years), adolescents (13–18 years), patients with advanced malignant conditions
Intervention/Indicator	Needs assessment tools for identifying palliative care needs	Educational tools or tools used to assess only one domain of the patient’s overall health status, such as pain, delirium, or quality of life
Comparison	Not required. Reviews may focus on one or more tools; direct comparisons between tools were included where available, but not mandatory.	
Outcome measures	Identifying needs in palliative care	Identifying needs, general and not specifically for palliative care
Time	Published within the last 10 years (2014–2024)	
Setting	All healthcare settings	
Study characteristics	Systematic reviews and meta-analyses (secondary material).	Systematic review protocol, evaluation of poor quality (lacking a study selection flowchart and explicit inclusion/exclusion criteria), and grey literature.
Language of publication	Abstract in English, full text in English or Greek.	

**Table 2:** Search strategy Database: Cochrane Library, from inception up to 30 June 2025.

Search ID	Search Term	Results
#1	MeSH descriptor: [Palliative Care] explode all trees	2282
#2	MeSH descriptor: [Hospice Care] explode all trees	163
#3	MeSH descriptor: [Hospice and Palliative Care Nursing] explode all trees	78
#4	MeSH descriptor: [Advance Care Planning] explode all trees	429
#5	MeSH descriptor: [Terminal Care] explode all trees	707
#6	MeSH descriptor: [Health Services Needs and Demand] explode all trees	631
#7	MeSH descriptor: [Needs Assessment] explode all trees	480
#8	MeSH descriptor: [Systematic Review] explode all trees	426
#9	MeSH descriptor: [Meta-Analysis as Topic] explode all trees	1460
#10	(meta analys *): ti,ab,kw OR (meta synthes *): ti,ab,kw OR (systematic review *): ti,ab,kw OR (review *): ti,ab,kw	5546
#11	(Diagnostic tool *): ti,ab,kw OR (Identification tool *): ti,ab,kw OR (Needs assessment tools *): ti,ab,kw OR (Instruments): ti,ab,kw	884
#12	(#1 OR #2 OR #3 OR #4 OR #5) AND (#6 OR #7 OR #11)	22
#13	#12 AND (#8 OR #9 OR #10)	5

The asterisk (*) indicates a truncation symbol used to capture variations of the root term.

**Table 3:** Overview of the included reviews and their methodological characteristics.

Authors, Year, Journal	Study Design And Aim	Number and Name of Searched Databases	Search Period	Number of Included Studies	Number and Name of Included Needs Assessment Tools *	Psychometric Properties of Needs Assessment Tools
[32]; BMJ Supportive & Palliative Care	Study design: systematic literature review Aim: To document what tools to use to identify patients with palliative care needs are available in the published literature, and to ascertain how GPs in Europe currently identify patients for palliative care	2 (PubMed, Embase)	From Inception up to the end of April 2012	5	7 (NECPAL; GSF-PIG; SPICT; RADPAC; Residential home palliative care tool; Rainone; QUICK GUIDE)	NR
[39]; Palliative Medicine	Study design: Systematic review and narrative synthesis Aim: To synthesize evidence on the end-of-life care needs of people with frailty.	14 (CINAHL, Cochrane, Embase, EThOS, Google, Medline, NDLTD, NHS Evidence, NICE, Open grey, PsycINFO, SCIE, SCOPUS, Web of Science	From inception up to October 2017	20	4 (CSHA-CFS; GSF frailty criteria; NECPAL; RAI)	NR
[40]; Palliative Medicine	Study design: Systematic review Aim: To identify existing needs assessment tools for the identification of patients with advanced non-malignant chronic conditions who are likely to have Palliative care needs in primary healthcare and evaluate their accuracy.	4 (Cochrane, MEDLINE, Embase, and CINAHL)	From inception up to March 2019	8	10 (SPICT; NECPAL; RADPAC; GSF-PIG; PALLI; SQ; The double SQ; AnticiPal electronic tool; Racine tool; eFI)	NR
[41]; Heart Failure Reviews	Study design: systematic mixed-studies review with narrative synthesis *Aim*: To identify the most appropriate palliative care needs-assessment/measurement tools for patients with heart failure	6 (Cochrane Library, MEDLINE Complete (EBSCO), AMED (EBSCO), PsycINFO (EBSCO), CINAHL Complete (EBSCO), and EMBASE (Ovid)	From inception up to 25 June 2020	27	6 (IPOS; GSF-PIG; RADPAC; SPICT; NAT:PD-HF; NECPAL)	They are reported
[42]; Academic Emergency Medicine	Study design: Systematic review Aim: To identify and assess the psychometric properties of the available needs assessment tools to identify patients with potential palliative care needs in the emergency department (ED).	7 (OVID Medline, Ovid EMBASE, OVID Health and Psychosocial Tools, EBSCO-CINAHL, SCOPUS, ProQuest Dissertations and Theses Global, Cochrane Library, and PROSPERO)	Searches were last updated in August 2021	35	12 (SQ and Modified SQ; P-CaRES and Modified P-CaRES; The SPEED tool and The 5-item SPEED tool; Palliative care trigger tool; Battery of tests including: NEST-13, ESAS, MQOL Questionnaire, Two-stage BriefPal screening protocol; IPAL-EM screening tool; Palliative Screening Tool (Unamed); 7-item Palliative care screening; SST; The Criteria for Receiving Palliative Care; Palliative Care Tool -Unamed; A-qCPR risk score)	They are reported
[43]; BMC Palliative Care	Study design: Systematic review and narrative synthesis Aim: to identify and synthesize eligibility criteria for trials in palliative care to construct a needs-based set of triggers for timely referral to palliative care for older adults severely affected by advanced non-malignant chronic conditions.	6 (MEDLINE (Ovid), EMBASE (Ovid), CINAHL (EBSCOhost), PsycINFO (Ovid), the Cochrane Central Register of Controlled Trials (CENTRAL), and Clinical- Trials.gov)	From inception up to June 2022	27	6 (PPS; KCCQ; FAST; PC-NAT; CSHA-CFS; SQ)	NR
[44]; BMJ Supportive & Palliative Care	Study design: Systematic review and meta-analysis Aim: (1) to identify the screening tools used by health professionals to promote the early identification of patients who may benefit from palliative care and (2) to assess the psychometric properties and clinical performance of the tools.	6 Four English databases (PubMed, CINAHL, Embase, Scopus) and two Chinese databases (CNKI and Wanfang)	From inception up to May 2023.	31	7 (GSF-PIG; RADPAC; TW-PCST; NECPAL; SPICT; Rainone; AnticiPal)	They are reported

* A-qCPR: Admission Quick Sequential Organ Failure Assessment for the Chronic Palliative Risk, CSHA-CFS: Canadian Study of Health and Aging-Clinical Frailty Scale, ESAS: Edmonton Symptom Assessment Scale, eFI: electronic Frailty Index, FAST: Functional Assessment Staging Test, GSF-PIG: Gold Standard Framework–Proactive Identification Guidance, IPAL-EM: Improving Palliative Care in Emergency Medicine, IPOS: Integrated Palliative care Outcome Scale, KCCQ: Kansas City Cardiomyopathy Questionnaire, MQOL: McGill Quality of Life Questionnaire, NAT:PD-HF: Needs Assessment Tool: Progressive Disease—Heart Failure, NECPAL: NECesidades Paliativas, NEST: Needs at the End-of- life, NR: Not Reported, P-CaRES: Palliative care and rapid emergency screening, PALLI: PALliative care: Learning to Identify in people with intellectual disabilities, PC-NAT: Palliative Care Needs Assessment Tool, PPS: Palliative Performance Scale, RADPAC: The RADboud indicators for PAlliative Care Needs, RAI: Risk analysis index, SPEED: Screening for palliative and end-of-life care needs in the emergency department, SPICT: Supportive & Palliative Care Indicators Tool, SST: Simplified Screening Tool, SQ: Surprise Question, TW-PCST: Taiwanese version-Palliative Care Screening Tool.

**Table 4:** Characteristics of the identified needs assessment tools (*n* = 35).

Needs Assessment Tools/Year and Country of Development/Available Language Versions	Original Reference	Target Population	Scope	Setting	Type/ Form (Paper-Based/Electronic Tool)	Completed by	Action/ Follow-Up Section Included?	Average Time for Completion	Indicators or Themes/Domains	Inclusion SQ	Criteria for Palliative Care/ Cutoff Value
FAST/ 1988, USA/English	[45]	Dementia (end-stage)	Disease-specific	Hospice settings	Paper-based	Healthcare professionals, Carers, and Family members	No—Purely observational or scoring tools.	NR	functional abilities, including physical (dressing and grooming), language (memory and recognition), mobility or self-feeding and other tasks	NO	No cutoff point. Generally, a FAST score of 7A (Stage 7A: Speech limited to about half a dozen intelligible words) or higher indicates end-stage dementia, requiring end-of-life care.
ESAS/ 1991, Canada/ English, Spanish, Korean, Italian, Turkish, Japanese, Portuguese, Chinese, French, Danish, German, Hungarian, Icelandic, Hebrew, Russian, Arabic, Dutch, Polish, Swedish, Thai, English Afrikaans	[46]	Multiple advanced chronic conditions	General	Multiple sites	Paper-based	Healthcare professionals, Patients, Family caregivers	No—Used for symptom tracking; no action/follow-up documentation built in.	Less than 2 min	Severity of physical and mental symptoms of distress	NO	No cutoff point. Provides a clinical profile of symptom severity over time.
MQOL Questionnaire/ 1995, Canada/ English and translated into more than 20 languages, among which Spanish, French, and Chinese	[47]	Life-threatening illness (any condition)	General	Multiple sites	Paper-based	Healthcare professionals	No—Focused on quality-of-life measurement only.	NR	Self-rating QOL on a scale of 0–10, with four subscales: physical symptoms, psychological symptoms, outlook on life, meaningful existence	NO	NR
PPS/ 1996, Canada/ Arabic, Catalan, Chinese, Czech, Dutch, English, Estonia, French, German, Greek, India, Indonesia, Japanese, Polish, Portuguese, Spanish, Thai, Turkish	[48]	Advanced cancer, dementia, frailty, etc.	General	Long-term care (LTC), Hospital	Paper-based	Registered staff to PSWs, Nurses, Physicians, Respiratory Therapists, Physiotherapists, Occupational Therapists, dieticians, pastoral, social workers, counselors, volunteers, family, patients	No—Purely observational or scoring tools.	NR exactly (The tool is quick and easy to use)	Five functional dimensions: ambulation, activity level, evidence of disease, self-care, oral intake, and level of consciousness	NO	11 levels (0–100% in 10% increments). A PPS score of 70% or below may indicate hospice eligibility.
KCCQ/ 2000, USA/Spanish French German Italian, Portuguese Dutch Japanese Chinese (Mandarin) Korean Turkish	[49]	Heart Failure	Disease-specific	Multiple sites	Paper-based	Healthcare professionals, Patients	No—Purely observational or scoring tools.	NR	Physical limitations, symptoms (frequency, severity, change over time), social limitations, self-efficacy, quality of life	NO	No cutoff point. Scores from 0–100, summarized in 25-point ranges (0–24: very poor to poor; 25–49: poor to fair; 50–74: fair to good; 75–100: good to excellent).
NEST-13/ 2001, USA/ English	[50]	Multiple comorbidities	General	Clinical settings, Emergency department	Paper-based	Healthcare professionals, Patients	Possibly—Highlights needs across domains; no explicit action section, but designed to guide care discussions.	NR	Thirteen domains: financial, access to care, caregiving, illness distress, physical health, mental health, closeness, spirituality, settledness, purpose, patient/provider communication, information, goals of care	NO	No cutoff point. Aims to assist physicians in identifying specific patient needs
GSF-PIG/ 2003, UK/English, Italian	[51]	Cancer, CHF, COPD, frailty	Mixed	Multiple sites	Paper-based	Healthcare professionals	No—Includes SQ and indicators but lacks a dedicated action section.	NR	73 indicators: 12 general, 12 sets of specific indicators	YES	SQ+, ≥1 general indicator or ≥1 specific indicator)
Racine tool/ 2004, Canada/ English	[52]	All *	General	Primary care	Electronic tool	Healthcare professionals		NA	NR	NR	Patient included if electronic records contain at least one high-risk death marker within the next year (e.g., age > 75, diagnosis of congestive heart failure)
CSHA-CFS/ 2005, Canada/ Arabic, Czech, Chinese, Danish, Dutch, Estonian, Finnish. French, German, Greek, Italian, Japanese, Korean, Latvian, Lithuanian, Norwegian, Polish, Portuguese, Slovene, Spanish, Swedish, Turkish	[53]	Frailty (65+)	Disease-specific	Hospital/Emergency department	Paper-based	Healthcare professionals	No—Purely observational or scoring tools.	Less than 2 min	70 deficits, including presence and severity of current diseases, ability in ADLs, physical signs from clinical and neurologic exams. Specific domains: comorbidity, function, cognition, generating a frailty score	NO	No cutoff point. The highest grade of the CFS (level 9), incorporated both severe frailty and terminal illness
Rainone/ 2007, USA/ NA	[54]	All *	General	General practice	Electronic tool	Healthcare professionals	No—Includes SQ and other risk factors but no formal follow-up documentation.	NR	6 indicators in total	YES	The SQ response was ‘No’ and/or answer affirmatively to any of items 2~5
SQ/ 2008, USA/ English, Italian	[55]	All *	General	Primary care, Hospital	Paper-based	Healthcare professionals	No—A single prognostic question; no action mechanism.	NR (one question)	NA	YES	Answer “no” to the ‘surprise’ question
PC-NAT/ 2008, USA/ English	[56]	Cancer, Parkinson’s disease	Disease-specific	Multiple sites (generalist abd specialist care)	Paper-based	Healthcare professionals	Possibly—Focuses on referral and family needs but unclear if it includes explicit post-assessment action plan.	NR	Patient well-being: Physical, Psychological, Spiritual. Caregiver/family ability to care for patient: Physical, Psychological, Family and relationships. Referral to SPCS	NO	NR
Palliative care trigger tool/ 2011, USA/ English	[57]	Elderly, multi-organ failure	General	Emergency department	Paper-based	Nurse practitioner	No—Identifies needs; no built-in action tracking.	NR (Three questions)	Three questions include: (1) “Does this patient have a progressive incurable illness that is in its later stages” (2) Do you know if the patient is expected to die on this hospital admission? (3) Would you be surprised if this patient were to die in the next year? Additional items assessing the medical necessity for palliative care and the need for advanced care planning	YES	Positive response (one or more) to any triggers indicates potential palliative care needs
SPEED (original version and a modified version-The 5-item SPEED)/ 2011, USA/ English	[58]	Advanced HF, cancer	General	Emergency department	Paper-based	Healthcare professionals	Yes—Especially in its modified version, includes practical care planning elements (e.g., pain management, psychological support).	NR	Original version: 13 items (social, therapeutic, physical, psychological, spiritual needs). Modified version: 5 items (pain management, home care, medication management, psychological support, goals of care): 5 items regarding difficulties in pain management, home care, medication management, psychological support, and goals of care	NO	No cutoff point. Aims to assist physicians in identifying specific patient needs.
Two-stage BriefPal screening protocol/ 2011, USA/ English	[59]	Elderly with life- threatening conditions **	General	Emergency department	Paper-based	Healthcare professionals, Palliative specialists	Possibly—Second stage includes care planning tools, though no structured documentation section reported.	NR	Initial Screening Stage and Comprehensive Assessment Stage. Needs assessment tools: Karnofsky Performance Scale Index, Functional Assessment Staging Tool, Memorial Symptom Assessment Scale, Katz ADL Scale, Brief Assessment Scale for Caregivers	NO	Karnofsky score < 80 or loss of ADLs identified as in need of palliative consultation.
QUICK GUIDE/ 2011, UK/ English	[60]	Various chronic diseases	Mixed	Primary care	Paper-based	GPs	No—Designed for early identification; no follow-up or care planning section included.	NR	General indicators (functional status, weight loss, hospital admissions) and disease-specific indicators	YES	Functional status: In bed >50% of the day MRC breathlessness scale 4/5 (REF), NYHA grade 3/4 (REF), WHO performance grade 3/4 (REF) Weight loss: >10% in 3–6 months Hospital admissions: >2 in the last 6 months Other: One or more life-threatening illness Burden of illness (physical, psychological, financial, other)
Palliative screening tool (Unnamed)/ 2011, USA/ English	[61]	Multiple advanced conditions	General	Emergency department	NR	Healthcare professionals	No—Screening-focused; lacks formal follow-up documentation.	NR	NR	NR	Patients with one or more triggers identified by an ED physician are considered to have potential palliative care needs.
RADPAC/ 2012, The Netherlands/ Dutch	[62]	CHF, COPD, cancer	General	Primary care/general practice	Paper-based	Primary care practitioners	No—Purely observational or scoring tools.	NR	21 indicators in 3 categories: Disease-specific indicators (COPD, CHF, Cancer), General indicators, SQ, functional status), Indicators of declining health status (psychosocial and caregiver indicators)	NO	No cutoff point
NECPAL/ 2013, Spain/English, Spanish, Portugues, Israeli	[63]	Multiple advanced chronic conditions	Mixed	Multiple sites	Paper-based	Healthcare professionals	No—Acts as early identification; no formal action section, though may guide care planning.	Older version: 2–8 min	59 indicators: 12 general, 9 sets of specific indicators	YES	SQ+, and ≥1 general indicator or ≥1 specific indicator
IPAL-EM screening tool/ 2013, USA/ NA	[64]	Life-threatening conditions **	General	Emergency department	Paper-based	Healthcare professionals	No—Identifies needs; no built-in action tracking.	NA	Symptom burden, functional status, psychosocial concerns, goals of care	NR	No cutoff point.
NAT: PD-HF/ 2013, USA/ English, Dutch, German	[33]	Heart Failure	Disease-specific	Multiple sites	Paper-based	Healthcare professionals	Yes—Includes a specific section prompting documentation of actions taken after assessment.	5–10 min. (Dutch version: 26 min)	30-item survey assessing needs across four subscales: physical, psychological, social, and existential.	NO	NR
SPICT/ 2014, UK/English, Thai, Spanish, Italian, German, Swedish, Indonesian, Danish, Japanese, Nepali, Dutch, French, Greek, Portuguese	[31]	Cancer, Heart/vascular disease, Kidney disease, Dementia/frailty, Respiratory disease, Liver disease, Neurological disease	Mixed	Multiple sites	Paper-based	Healthcare professionals	No—Primarily identification; follow-up left to clinician’s discretion.	SPICT: A few minutes. SPICT-LIS: An average of 3.3 min; SPICT-ES: An average of 4 min and 45 s.; SPICT-DE: An average of 7.5 min	34 indicators: 6 general, 23 specific clinical, 5 recommended	NO	≥2 general indicators and ≥1 clinical indicators
7-item Palliative care screening/ 2015, USA/ English	[65]	Advanced conditions, frequent admissions	General	Emergency department	NR	NR	No—Risk-based identification only; follow-up process not included.	NR	NR	NR	Patients with one or more risk factors were identified as having potential palliative care needs.
P-CaRES tool (original version and modified version)/ 2015, USA/English	[66]	End-stage chronic conditions	Mixed	Emergency department	Paper-based	Healthcare professionals	Possibly—Includes criteria that imply action, but not a structured follow-up documentation section.	NR (two questions)	The first domain asks physicians to identify at least one end-of-life condition. The second domain includes frequent hospital visits, uncontrolled symptoms, functional decline, uncertainty about goals of care, caregiver distress, and the SQ (“You would not be surprised if this patient died within 12 months”).	YES	Patients with at least one EOL condition and two or more risk factors for potential palliative care needs were identified as having palliative care needs.
eFI/ 2016, UK/ NA	[67]	All *	General	Primary care	Electronic tool	GPs	No—Electronic frailty score; does not include any structured care planning section.	NA	ΝA	NO	No cutoff point. It presents an output as a score indicating the number of deficits present out of a possible total of 36, with higher scores indicating an increasing likelihood of a person living with frailty and, hence, vulnerability to adverse outcomes.
RAI/ 2017, USA/ English	[68]	Frailty of the surgical patient	Disease-specific	Hospital	Paper-based	Healthcare professionals	No—Scoring tool only; does not include post-assessment action tracking.	NR	14-item tool assessing deficits across five domains of frailty (physical, functional, social, nutritional, and cognitive).	NO	The RAI score ranges from 0 to 81, and a cutoff of 30 was chosen based on prior work that identified this value as optimal for maximizing negative predictive value.
SST/ 2017, Italy/ English	[69]	chronic organ failure (i.e., heart, lungs, liver, and kidneys), progressive neurological diseases (i.e., dementia, stroke, Parkinson’s disease, ALS, MS, and advanced cancer	General	Emergency department	Paper-based	Healthcare professionals	No—Applies inclusion criteria; no documentation section for interventions.	NR	The first criterion refers to the PPS score (0–100). The second criterion refers to the presence of at least one of six clinical indicators, including ≥1 admission within the last 12 months; hospital admission from healthcare services; awaiting admission to long-term care, healthcare services, or hospice; dialysis; home oxygen use; or non-invasive ventilation.	YES	When the Palliative Performance Scale < 50 is present with at least one of these indicators: ≥1 admission within the last 12 months; hospital admission from HCS; awaiting admission to HCS/Hospice; dialysis; home oxygen use; non-invasive ventilation.
Anticipate/ 2018, UK/ English	[70]	All *	General	Primary care/General practice	Electronic tool	Healthcare professionals	Possibly—Electronic flags may trigger actions, but no structured follow-up documentation was reported.	NR	NR	NA	if one or more Inclusion criteria are met, None of the exclusion criteria are met. The Inclusion criteria: Type 1: Malignancy codes, e.g., pancreatic cancer. Type 2: Other single Read Codes at any time, e.g., Frailty. Type 3: Combinations of Read Codes, e.g., difficulty swallowing and dementia
PALLI/ 2018, The Netherlands/ Dutch, English	[71]	Intellectual disabilities	Disease-specific	Primary care	Paper-based	Healthcare professionals	Possibly—Used to inform decisions in intellectual disability care; unclear whether structured follow-up is recorded.	The mean time of 10.5 min (physicians) and 10.1 min (daily care professionals)	39 items categorized into nine themes (physical, activities, characteristic behavior, statements of people with intellectual disabilities and family regarding decline, signs and symptoms, recurrence of infections, frailty, serious illnesses, and prognosis).	NO	No cutoff point
IPOS (original version), IPOS Neuro, IPOS-Dem, IPOS-Renal, IPOS-HF/ 2019, UK, German/ Arabic, Czech, Danish, English, Estonian, French, Greek, Hindi, Italian, Korean, Malay, Myanmar, Persian, Polish, Portuguese, Singapore, Swedish, Turkish	[72]	Various advanced conditions	General	Multiple sites	Paper-based	Healthcare professionals (staff version), Patients and carers (patient version)	No—Does not include a dedicated action section; results are often used but not directly tied to documented clinical response.	Staff version: 2–5 min Patient version: 8 min	10 questions scored on a scale of 1–4 assessing physical, social, psychological, and spiritual needs	NO	The overall IPOS score ranges from zero to 68. The IPOS score is useful for understanding the patient’s symptoms, concerns, and status at a specific point in time.
double SQ/ 2019, The Netherlands/ Dutch, Slovak	[73]	All *	General	Primary care, Hospital	Paper-based	Healthcare professionals	No—Prognostic only; no care planning or follow-up documentation.	NR (two questions)	NA	YES	a combination of SQ1: ‘no’ and SQ2: ‘yes’
The Criteria for Receiving Palliative Care/ 2020, Turkey/ Turkish	[74]	Multiple life-threatening conditions **	General	Emergency department	Paper-based	Healthcare professionals	No—Scoring-based eligibility tool; does not include an action section.	NR	The four components include: Main Disease Criteria (cancer, advanced COPD, stroke, terminal kidney failure, advanced heart failure, other diseases and conditions that shorten lifespan); Existence of accompanying disease; Determination of patients’ functionality; Applicability of other criteria prepared by the Provincial Directorate of Health.	NR	Patients who scored 3 or higher according to this form were considered as palliative care patients (PCP)
Palliative Care Tool-Unamed/ 2020, Peru/ English	[75]	Advanced disease	General	Emergency department	Paper-based	ED physicians	No—Includes SQ and palliative care indicators, but no follow-up action section mentioned.	NR	7 questions: The SQ: “Would you be surprised if this patient died within one year?”; knowledge of palliative care; need for palliative care; request for PC; use of palliative care; symptom control; presence of caregiver.	YES	A ‘Yes’ response to Question 3 indicated a patient with potential palliative care needs.
A-qCPR/ 2021, USA/ NA	[76]	Advanced chronic conditions	General	Emergency Department/Hospital	Paper-based	Healthcare professionals	No—Purely predictive risk score; no post-assessment component.	NR (takes only a few minutes to complete)	Consists of 5 risk factors for mortality: Age (0.05 points per year) (2 points); qSOFA score of 2 or more (1 point); performance score of two or more (2 points); Had DNR (3 points); Had Cancer (4 points).	NA	A score of 9 or more indicates patients with potential palliative care needs.

A-qCPR: Admission Quick Sequential Organ Failure Assessment for the Chronic Palliative Risk, ADL: activity of daily living, ALS: amyotrophic lateral sclerosis, CHF: Chronic Heart Failure, CNS: central nervous system, COPD: Chronic Obstructive Pulmonary Disease, CSHA-CFS: Canadian Study of Health and Aging-Clinical Frailty Scale, ECOG: Eastern Cooperative Oncology Group, eFI: electronic Frailty Index, EOL: end-of-life, ESAS: Edmonton Symptom Assessment Scale, FAST: Functional Assessment Staging Test, GPS: General Practitioners, GSF-PIG: Gold Standard Framework–Proactive Identification Guidance, HCS: health care services, IPAL-EM: Improving Palliative Care in Emergency Medicine, IPOS: Integrated Palliative care Outcome Scale, KCCQ: Kansas City Cardiomyopathy Questionnaire, MND: Motor Neurone Disease, MQOL: McGill Quality of Life Questionnaire, NA: Not Applicable, NAT:PD-HF: Needs Assessment Tool: Progressive Disease—Heart Failure, NECPAL: NECesidades Paliativas, NEST: Needs at the End-of- life, NR: Not Reported, MS: Multiple Sclerosis, P-CaRES: Palliative care and rapid emergency screening, PALLI: PALliative care: Learning to Identify in people with intellectual disabilities, PC-NAT: Palliative Care Needs Assessment Tool, PSWs: Personal support workers, PPS: Palliative Performance Scale, RADPAC: The RADboud indicators for PAlliative Care Needs, RAI: Risk analysis index, SPCS: specialist palliative care service, SPEED: Screening for palliative and end-of-life care needs in the emergency department, SST: Simplified Screening Tool, SPICT: Supportive & Palliative Care Indicators Tool, SQ: Surprise Question, TW- PCST: Taiwanese version-Palliative Care Screening Tool. * All: patients with a specific clinical condition it including heart failure, advanced cancer, COPD, and sepsis, patients without a specific clinical condition, patients who were to be admitted to the hospital, admitted to the intensive care unit (ICU) or any patients older than 65 or 70 years; ** Life-threatening conditions: is defined as any disease/disorder known to be life-limiting (e.g., dementia, COPD, metastatic cancer) or that has a high chance of leading to death (e.g., multi-organ failure, sepsis).

**Table 5:** Summary classification of needs-assessment tools by primary purpose.

Category	Tools (*n* = 35)
Early-identification tools	GSF-PIG; SPICT; QUICK GUIDE; NECPAL; RADPAC; SQ; double SQ; Palliative Care Trigger Tool; Rainone tool; A-qCPR; Criteria for Receiving Palliative Care (Turkey); SST; Two-stage BriefPal (Screening Stage); 7-item Palliative Care Screening Tool; Palliative Screening Tool (Unnamed, USA); Anticipate; eFI.
Comprehensive multidomain assessment tools	PC-NAT; NAT:PD-HF; SPEED (original and 5-item version); IPOS (all versions).
Mixed-purpose tools (tools assessing functional status, frailty, QoL, or combining screening with partial needs assessment)	PPS; KCCQ; NEST-13; CSHA-CFS; RAI; TW-PCST; IPAL-EM; Racine Tool; P-CaRES (original and modified versions); PALLI; FAST; ESAS; MQOL Questionnaire.

**Table 6:** Summary of reported psychometric properties of the needs assessment tools.

Reference of Systematic Review	Needs Assessment Tool	Reliability	Validity			
			Content Validity	Criterion Validity	Construct Validity	Face Validity
[41]	NAT: PD-HF (original)	good	good	good	good	good
	NAT:PD-HF (Dutch)	NR	NR	poor	poor	NR
[42]	13-item SPEED	good ^1^	NR	NR	NR	NR
	SQ	NR	NR	NR	doubtful ^2^	NR
	Palliative Care Tool-Unamed (Peru)	good ^3^	NR	NR	NR	NR
[44]	SPICT	good ^4^	NR	NR	NR	NR
	Italian-SPICT	NR	very good ^5^	NR	NR	NR
	Israeli-NECPAL	NR	very good ^6^	NR	NR	NR

NAT: PD-HF: Needs Assessment Tool: Progressive Disease—Heart Failure, NECPAL: NECesidades Paliativas, NR: Not Reported, SPICT: Supportive & Palliative Care Indicators Tool, SQ: Surprise Question, ^1^: Cronbach coefficient for survey scales (0.716–0.991), ^2^: Moderate-strong correlation between SQ and Caregiver Burden Score-CBS (r = −0.35) and Risk Instrument for Screening in the Community-RISC (r = −0.68). Poor correlation between SQ and CFS and EQ-5D (<0.1), ^3^: Cronbach’s coefficient = 0.699, ^4^: a range of 0.35–0.97 on the Kuder-Richardson formula, a Kappa range of 0.66–0.98, and a Cronbach’s alpha of 0.84, ^5^ for the Italian-SPICT, which was 0.86. ^6^ a Kappa-adjusted of 0.96 for the Israeli-NECPAL.

## Data Availability

No new data were created or analyzed in this study.

## Supplementary Materials

The following supporting information can be downloaded at https://www.mdpi.com/article/10.3390/healthcare14010046/s1. Table S1: PRIOR Checklist (Preferred Reporting Items for Overviews of Reviews); Table S2: Search strategy Database: EBSCO host, CINAHL from inception to 30 June 2025; Table S3: Search strategy Database: PubMed from inception to 30 June 2025; Table S4: Search strategy Database: PsycINFO from inception to 30 June 2025; Table S5: Risk of Bias Assessment of Included Systematic Reviews; Table S6: Potential Appraisal Tools Considered for Future Umbrella Reviews (COSMIN RoB and QUADAS-2).

## Registration

PROSPERO (CRD42024553053).

## Abbreviations

The following abbreviations are used in this manuscript:

ADL	Activities of Daily Living
A-qCPR	Admission Quick Sequential Organ Failure Assessment for the Chronic Palliative Risk
ALS	Amyotrophic Lateral Sclerosis
COPD	Chronic Obstructive Pulmonary Disease
COSMIN	COnsensus-based Standards for the selection of health Measurement INstruments
CSHA-CFS	Canadian Study of Health and Aging–Clinical Frailty Scale
ED	Emergency Department
EDs	Emergency Departments
eFI	Electronic Frailty Index
ESAS	Edmonton Symptom Assessment Scale
FAST	Functional Assessment Staging Test
GSF-PIG	Gold Standards Framework–Proactive Identification Guidance
HF	Heart Failure
IPOS	Integrated Palliative care Outcome Scale
JBI	Joanna Briggs Institute
KCCQ	Kansas City Cardiomyopathy Questionnaire
MQOL	McGill Quality of Life Questionnaire
NAT: PD-HF	Needs Assessment Tool: Progressive Disease–Heart Failure
NECPAL	NECesidades Paliativas
PC	Palliative Care
PC-NAT	Palliative Care Needs Assessment Tool
PPS	Palliative Performance Scale
PRIOR	Preferred Reporting Items for Overviews of Reviews
PRISMA	Preferred Reporting Items for Systematic Reviews and Meta-Analyses
PROSPERO	International Prospective Register of Systematic Reviews
QUADAS-2	Quality Assessment of Diagnostic Accuracy Studies 2
RAI	Risk Analysis Index
SPEED	Screening for Palliative and End-of-life care needs in the Emergency Department
SPICT	Supportive and Palliative Care Indicators Tool
SQ	Surprise Question
SST	Simplified Screening Tool
TW-PCST	Taiwanese version–Palliative Care Screening Tool

## References

[1] Patel P., Lyons L. (2020). Examining the Knowledge, Awareness, and Perceptions of Palliative Care in the General Public Over Time: A Scoping Literature Review. Am. J. Hosp. Palliat. Care.

[2] Tanuseputro P., Wodchis W.P., Fowler R., Walker P., Bai Y.Q., Bronskill S.E., Manuel D. (2015). The Health Care Cost of Dying: A Population-Based Retrospective Cohort Study of the Last Year of Life in Ontario, Canada. PLoS ONE.

[3] Lang J.J., Alam S., Cahill L.E., Drucker A.M., Gotay C., Kayibanda J.F., Kozloff N., Mate K.K.V., Patten S.B., Orpana H.M. (2018). Global Burden of Disease Study Trends for Canada from 1990 to 2016. CMAJ.

[4] Kavalieratos D., Corbelli J., Zhang D., Dionne-Odom J.N., Ernecoff N.C., Hanmer J., Hoydich Z.P., Ikejiani D.Z., Klein-Fedyshin M., Zimmermann C. (2016). Association Between Palliative Care and Patient and Caregiver Outcomes: A Systematic Review and Meta-Analysis. JAMA.

[5] McIlvennan C.K., Allen L.A. (2016). Palliative Care in Patients with Heart Failure. BMJ.

[6] Worldwide Hospice Palliative Care Alliance, World Health Organization (2020). Global Atlas of Palliative Care.

[7] Lunney J.R. (2003). Patterns of Functional Decline at the End of Life. JAMA.

[8] Seow H., O’Leary E., Perez R., Tanuseputro P. (2018). Access to Palliative Care by Disease Trajectory: A Population-Based Cohort of Ontario Decedents. BMJ Open.

[9] Chidiac C. (2018). The Evidence of Early Specialist Palliative Care on Patient and Caregiver Outcomes. Int. J. Palliat. Nurs..

[10] Knaul F.M., Farmer P.E., Krakauer E.L., De Lima L., Bhadelia A., Jiang Kwete X., Arreola-Ornelas H., Gómez-Dantés O., Rodriguez N.M., Alleyne G.A.O. (2018). Alleviating the Access Abyss in Palliative Care and Pain Relief—An Imperative of Universal Health Coverage: The Lancet Commission Report. Lancet.

[11] Kwete X.J., Bhadelia A., Arreola-Ornelas H., Mendez O., Rosa W.E., Connor S., Downing J., Jamison D., Watkins D., Calderon R. (2024). Global Assessment of Palliative Care Need: Serious Health-Related Suffering Measurement Methodology. J. Pain. Symptom Manag..

[12] Moens K., Higginson I.J., Harding R., Brearley S., Caraceni A., Cohen J., Costantini M., Deliens L., Francke A.L., Kaasa S. (2014). Are There Differences in the Prevalence of Palliative Care-Related Problems in People Living with Advanced Cancer and Eight Non-Cancer Conditions? A Systematic Review. J. Pain. Symptom Manag..

[13] Currow D.C., Allingham S., Bird S., Yates P., Lewis J., Dawber J., Eagar K. (2012). Referral Patterns and Proximity to Palliative Care Inpatient Services by Level of Socio-Economic Disadvantage. A National Study Using Spatial Analysis. BMC Health Serv. Res..

[14] Gomes B., Calanzani N., Curiale V., McCrone P., Higginson I.J., De Brito M. (2013). Effectiveness and Cost-Effectiveness of Home Palliative Care Services for Adults with Advanced Illness and Their Caregivers. Cochrane Database Syst. Rev..

[15] Gaertner J., Siemens W., Meerpohl J.J., Antes G., Meffert C., Xander C., Stock S., Mueller D., Schwarzer G., Becker G. (2017). Effect of Specialist Palliative Care Services on Quality of Life in Adults with Advanced Incurable Illness in Hospital, Hospice, or Community Settings: Systematic Review and Meta-Analysis. BMJ.

[16] Zimmermann C., Swami N., Krzyzanowska M., Hannon B., Leighl N., Oza A., Moore M., Rydall A., Rodin G., Tannock I. (2014). Early Palliative Care for Patients with Advanced Cancer: A Cluster-Randomised Controlled Trial. Lancet.

[17] Vanbutsele G., Pardon K., Van Belle S., Surmont V., De Laat M., Colman R., Eecloo K., Cocquyt V., Geboes K., Deliens L. (2018). Effect of Early and Systematic Integration of Palliative Care in Patients with Advanced Cancer: A Randomised Controlled Trial. Lancet Oncol..

[18] Bornais C., Deravin-Malone L. (2016). Early Palliative Care for Patients with Metastatic Lung Cancer: Evidence for and Barriers Against. Nurs. Palliat. Care.

[19] Temel J.S., Greer J.A., Muzikansky A., Gallagher E.R., Admane S., Jackson V.A., Dahlin C.M., Blinderman C.D., Jacobsen J., Pirl W.F. (2010). Early Palliative Care for Patients with Metastatic Non–Small-Cell Lung Cancer. N. Engl. J. Med..

[20] Allsop M.J., Ziegler L.E., Mulvey M.R., Russell S., Taylor R., Bennett M.I. (2018). Duration and Determinants of Hospice-Based Specialist Palliative Care: A National Retrospective Cohort Study. Palliat. Med..

[21] Janssen D.J., Boyne J., Currow D.C., Schols J.M., Johnson M.J., La Rocca H.-P.B. (2019). Timely Recognition of Palliative Care Needs of Patients with Advanced Chronic Heart Failure: A Pilot Study of a Dutch Translation of the Needs Assessment Tool: Progressive Disease—Heart Failure (NAT:PD-HF). Eur. J. Cardiovasc. Nurs..

[22] Rego F., Nunes R. (2019). The Interface Between Psychology and Spirituality in Palliative Care. J. Health Psychol..

[23] Bausewein C., Daveson B.A., Currow D.C., Downing J., Deliens L., Radbruch L., Defilippi K., Lopes Ferreira P., Costantini M., Harding R. (2016). EAPC White Paper on Outcome Measurement in Palliative Care: Improving Practice, Attaining Outcomes and Delivering Quality Services—Recommendations from the European Association for Palliative Care (EAPC) Task Force on Outcome Measurement. Palliat. Med..

[24] George N., Phillips E., Zaurova M., Song C., Lamba S., Grudzen C. (2016). Palliative Care Screening and Assessment in the Emergency Department: A Systematic Review. J. Pain. Symptom Manag..

[25] Walsh R.I., Mitchell G., Francis L., Van Driel M.L. (2015). What Diagnostic Tools Exist for the Early Identification of Palliative Care Patients in General Practice? A Systematic Review. J. Palliat. Care.

[26] Downar J., Wegier P., Tanuseputro P. (2019). Early Identification of People Who Would Benefit from a Palliative Approach—Moving From Surprise to Routine. JAMA Netw. Open.

[27] Richardson P., Greenslade J., Shanmugathasan S., Doucet K., Widdicombe N., Chu K., Brown A. (2015). PREDICT: A Diagnostic Accuracy Study of a Tool for Predicting Mortality within One Year: Who Should Have an Advance Healthcare Directive?. Palliat. Med..

[28] Boland J.W., Reigada C., Yorke J., Hart S.P., Bajwah S., Ross J., Wells A., Papadopoulos A., Currow D.C., Grande G. (2016). The Adaptation, Face, and Content Validation of a Needs Assessment Tool: Progressive Disease for People with Interstitial Lung Disease. J. Palliat. Med..

[29] Cardona-Morrell M., Hillman K. (2015). Development of a Tool for Defining and Identifying the Dying Patient in Hospital: Criteria for Screening and Triaging to Appropriate aLternative Care (CriSTAL). BMJ Support. Palliat. Care.

[30] Thomas K., Noble B. (2007). Improving the Delivery of Palliative Care in General Practice: An Evaluation of the First Phase of the Gold Standards Framework. Palliat. Med..

[31] Highet G., Crawford D., Murray S.A., Boyd K. (2014). Development and Evaluation of the Supportive and Palliative Care Indicators Tool (SPICT): A Mixed-Methods Study. BMJ Support. Palliat. Care.

[32] Maas E.A.T., Murray S.A., Engels Y., Campbell C. (2013). What Tools Are Available to Identify Patients with Palliative Care Needs in Primary Care: A Systematic Literature Review and Survey of European Practice. BMJ Support. Palliat. Care.

[33] Waller A., Girgis A., Davidson P.M., Newton P.J., Lecathelinais C., Macdonald P.S., Hayward C.S., Currow D.C. (2013). Facilitating Needs-Based Support and Palliative Care for People with Chronic Heart Failure: Preliminary Evidence for the Acceptability, Inter-Rater Reliability, and Validity of a Needs Assessment Tool. J. Pain. Symptom Manag..

[34] Aromataris E., Fernandez R., Godfrey C.M., Holly C., Khalil H., Tungpunkom P. (2015). Summarizing Systematic Reviews: Methodological Development, Conduct and Reporting of an Umbrella Review Approach. Int. J. Evid. Based Healthc..

[35] Bellali I., Miziou S., Dimitriadou-Maninia D. (2022). Typology and Methodology of Reviews in Health Research. Nurs. Care Res..

[36] Wiechula R., Conroy T., Kitson A.L., Marshall R.J., Whitaker N., Rasmussen P. (2016). Umbrella Review of the Evidence: What Factors Influence the Caring Relationship between a Nurse and Patient?. J. Adv. Nurs..

[37] Aromataris E., Lockwood C., Porritt K., Pilla B., Jordan Z. (2024). JBI Manual for Evidence Synthesis.

[38] Moher D., Liberati A., Tetzlaff J., Altman D.G., The PRISMA Group (2009). Preferred Reporting Items for Systematic Reviews and Meta-Analyses: The PRISMA Statement. PLoS Med..

[39] Stow D., Spiers G., Matthews F.E., Hanratty B. (2019). What Is the Evidence That People with Frailty Have Needs for Palliative Care at the End of Life? A Systematic Review and Narrative Synthesis. Palliat. Med..

[40] ElMokhallalati Y., Bradley S.H., Chapman E., Ziegler L., Murtagh F.E., Johnson M.J., Bennett M.I. (2020). Identification of Patients with Potential Palliative Care Needs: A Systematic Review of Screening Tools in Primary Care. Palliat. Med..

[41] Remawi B.N., Gadoud A., Murphy I.M.J., Preston N. (2021). Palliative Care Needs-Assessment and Measurement Tools Used in Patients with Heart Failure: A Systematic Mixed-Studies Review with Narrative Synthesis. Heart Fail. Rev..

[42] Kirkland S.W., Yang E.H., Garrido Clua M., Kruhlak M., Campbell S., Villa-Roel C., Rowe B.H. (2022). Screening Tools to Identify Patients with Unmet Palliative Care Needs in the Emergency Department: A Systematic Review. Acad. Emerg. Med..

[43] Kawashima A., Evans C.J. (2023). Needs-Based Triggers for Timely Referral to Palliative Care for Older Adults Severely Affected by Noncancer Conditions: A Systematic Review and Narrative Synthesis. BMC Palliat. Care.

[44] Xie Z., Ding J., Jiao J., Tang S., Huang C. (2024). Screening Instruments for Early Identification of Unmet Palliative Care Needs: A Systematic Review and Meta-Analysis. BMJ Support. Palliat. Care.

[45] Reisberg B. (1988). Functional Assessment Staging (FAST). Psychopharmacol. Bull..

[46] Bruera E., Kuehn N., Miller M.J., Selmser P., Macmillan K. (1991). The Edmonton Symptom Assessment System (ESAS): A Simple Method for the Assessment of Palliative Care Patients. J. Palliat. Care.

[47] Cohen S.R., Mount B.M., Strobel M.G., Bui F. (1995). The McGill Quality of Life Questionnaire: A Measure of Quality of Life Appropriate for People with Advanced Disease. A Preliminary Study of Validity and Acceptability. Palliat. Med..

[48] Anderson F., Downing G.M., Hill J., Casorso L., Lerch N. (1996). Palliative Performance Scale (PPS): A New Tool. J. Palliat. Care.

[49] Green C.P., Porter C.B., Bresnahan D.R., Spertus J.A. (2000). Development and Evaluation of the Kansas City Cardiomyopathy Questionnaire: A New Health Status Measure for Heart Failure. J. Am. Coll. Cardiol..

[50] Emanuel L.L., Alpert H.R., Emanuel E.E. (2001). Concise Screening Questions for Clinical Assessments of Terminal Care: The Needs Near the End-of-Life Care Screening Tool. J. Palliat. Med..

[51] Thomas K. (2003). The Gold Standards Framework in Community Palliative Care. Eur. J. Palliat. Care.

[52] Pereira J., Racine E. The Racine Tool: Identifying Patients for Palliative Care in Family Practice. Proceedings of the 15th International Congress on Care of the Terminally Ill.

[53] Rockwood K. (2005). A Global Clinical Measure of Fitness and Frailty in Elderly People. Can. Med. Assoc. J..

[54] Rainone F., Blank A., Selwyn P.A. (2007). The Early Identification of Palliative Care Patients: Preliminary Processes and Estimates from Urban, Family Medicine Practices. Am. J. Hosp. Palliat. Care.

[55] Moss A.H., Ganjoo J., Sharma S., Gansor J., Senft S., Weaner B., Dalton C., MacKay K., Pellegrino B., Anantharaman P. (2008). Utility of the “Surprise” Question to Identify Dialysis Patients with High Mortality. Clin. J. Am. Soc. Nephrol..

[56] Waller A., Girgis A., Currow D., Lecathelinais C. (2008). Development of the Palliative Care Needs Assessment Tool (PC-NAT) for Use by Multi-Disciplinary Health Professionals. Palliat. Med..

[57] Weissman D.E., Meier D.E. (2011). Identifying Patients in Need of a Palliative Care Assessment in the Hospital Setting a Consensus Report from the Center to Advance Palliative Care. J. Palliat. Med..

[58] Richards C.T., Gisondi M.A., Chang C.-H., Courtney D.M., Engel K.G., Emanuel L., Quest T. (2011). Palliative Care Symptom Assessment for Patients with Cancer in the Emergency Department: Validation of the Screen for Palliative and End-of-Life Care Needs in the Emergency Department Instrument. J. Palliat. Med..

[59] Glajchen M., Lawson R., Homel P., DeSandre P., Todd K.H. (2011). A Rapid Two-Stage Screening Protocol for Palliative Care in the Emergency Department: A Quality Improvement Initiative. J. Pain. Symptom Manag..

[60] McDaid P. (2011). A Quick Guide to Identifying Patients for Supportive and Palliative Care.

[61] Grudzen C.R., Stone S.C., Morrison R.S. (2011). The Palliative Care Model for Emergency Department Patients with Advanced Illness. J. Palliat. Med..

[62] Thoonsen B., Engels Y., Van Rijswijk E., Verhagen S., Van Weel C., Groot M., Vissers K. (2012). Early Identification of Palliative Care Patients in General Practice: Development of RADboud Indicators for PAlliative Care Needs (RADPAC). Br. J. Gen. Pr..

[63] Gómez-Batiste X., Martínez-Muñoz M., Blay C., Amblàs J., Vila L., Costa X., Villanueva A., Espaulella J., Espinosa J., Figuerola M. (2013). Identifying Patients with Chronic Conditions in Need of Palliative Care in the General Population: Development of the NECPAL Tool and Preliminary Prevalence Rates in Catalonia. BMJ Support. Palliat. Care.

[64] Quest T., Herr S., Lamba S., Weissman D. (2013). Demonstrations of Clinical Initiatives to Improve Palliative Care in the Emergency Department: A Report From the IPAL-EM Initiative. Ann. Emerg. Med..

[65] Schulman K.A., Zalenski R.J., Johnson C. (2015). Palliative Care Screening in the Emergency Department: 660. Acad. Emerg. Med..

[66] George N., Barrett N., McPeake L., Goett R., Anderson K., Baird J. (2015). Content Validation of a Novel Screening Tool to Identify Emergency Department Patients with Significant Palliative Care Needs. Acad. Emerg. Med..

[67] Clegg A., Bates C., Young J., Ryan R., Nichols L., Ann Teale E., Mohammed M.A., Parry J., Marshall T. (2016). Development and Validation of an Electronic Frailty Index Using Routine Primary Care Electronic Health Record Data. Age Ageing.

[68] Hall D.E., Arya S., Schmid K.K., Blaser C., Carlson M.A., Bailey T.L., Purviance G., Bockman T., Lynch T.G., Johanning J. (2017). Development and Initial Validation of the Risk Analysis Index for Measuring Frailty in Surgical Populations. JAMA Surg..

[69] Cotogni P., De Luca A., Evangelista A., Filippini C., Gili R., Scarmozzino A., Ciccone G., Brazzi L. (2017). A Simplified Screening Tool to Identify Seriously Ill Patients in the Emergency Department for Referral to a Palliative Care Team. Minerva Anestesiol..

[70] Mason B., Boyd K., Steyn J., Kendall M., Macpherson S., Murray S.A. (2018). Computer Screening for Palliative Care Needs in Primary Care: A Mixed-Methods Study. Br. J. Gen. Pr..

[71] Vrijmoeth C., Echteld M.A., Assendelft P., Christians M., Festen D., Van Schrojenstein Lantman-de Valk H., Vissers K., Groot M. (2018). Development and Applicability of a Tool for Identification of People with Intellectual Disabilities in Need of Palliative Care (PALLI). Res. Intellect. Disabil..

[72] Murtagh F.E., Ramsenthaler C., Firth A., Groeneveld E.I., Lovell N., Simon S.T., Denzel J., Guo P., Bernhardt F., Schildmann E. (2019). A Brief, Patient- and Proxy-Reported Outcome Measure in Advanced Illness: Validity, Reliability and Responsiveness of the Integrated Palliative Care Outcome Scale (IPOS). Palliat. Med..

[73] Veldhoven C.M.M., Nutma N., De Graaf W., Schers H., Verhagen C.A.H.H.V.M., Vissers K.C.P., Engels Y. (2019). Screening with the Double Surprise Question to Predict Deterioration and Death: An Explorative Study. BMC Palliat. Care.

[74] Bakan G., Ozen M., Azak A., Erdur B. (2020). Determination of the Characteristics and Outcomes of the Palliative Care Patients Admitted to the Emergency Department. Int. Emerg. Nurs..

[75] Amado Tineo J.P., Vasquez Alva R., Huari Pastrana R., Huamán Manrique P.Z., Oscanoa Espinoza T. (2020). Need for Palliative Care in Patients Admitted to Emergency Departments of Three Tertiary Hospitals: Evidence from a Latin-American City. Palliat. Med. Pr..

[76] Wang R.-F., Lai C.-C., Fu P.-Y., Huang Y.-C., Huang S.-J., Chu D., Lin S.-P., Chaou C.-H., Hsu C.-Y., Chen H.-H. (2021). A-qCPR Risk Score Screening Model for Predicting 1-Year Mortality Associated with Hospice and Palliative Care in the Emergency Department. Palliat. Med..

[77] Campbell R.T., Petrie M.C., Jackson C.E., Jhund P.S., Wright A., Gardner R.S., Sonecki P., Pozzi A., McSkimming P., McConnachie A. (2018). Which Patients with Heart Failure Should Receive Specialist Palliative Care?. Eur. J. Heart Fail..

[78] Mitchell G.K., Senior H.E., Rhee J.J., Ware R.S., Young S., Teo P.C., Murray S., Boyd K., Clayton J.M. (2018). Using Intuition or a Formal Palliative Care Needs Assessment Screening Process in General Practice to Predict Death within 12 Months: A Randomised Controlled Trial. Palliat. Med..

[79] Kane P.M., Ellis-Smith C.I., Daveson B.A., Ryan K., Mahon N.G., McAdam B., McQuillan R., Tracey C., Howley C., O’Gara G. (2018). Understanding How a Palliative-Specific Patient-Reported Outcome Intervention Works to Facilitate Patient-Centred Care in Advanced Heart Failure: A Qualitative Study. Palliat. Med..

[80] Sulmasy D.P. (2002). A Biopsychosocial-Spiritual Model for the Care of Patients at the End of Life. Gerontol..

[81] Walker Z.J., Xue S., Jones M.P., Ravindran A.V. (2021). Depression, Anxiety, and Other Mental Disorders in Patients with Cancer in Low- and Lower-Middle–Income Countries: A Systematic Review and Meta-Analysis. JCO Glob. Oncol..

[82] Cerullo G., Videira-Silva A., Carrancha M., Rego F., Nunes R. (2023). Complexity of Patient Care Needs in Palliative Care: A Scoping Review. Ann. Palliat. Med..

[83] Seven A., Sert H. (2023). Anxiety, Dyspnea Management, and Quality of Life in Palliative Care Patients: A Randomized Controlled Trial. FNJN.

[84] Batstone E., Bailey C., Hallett N. (2020). Spiritual Care Provision to End-of-life Patients: A Systematic Literature Review. J. Clin. Nurs..

[85] Fradelos E.C. (2021). Spiritual Well-Being and Associated Factors in End-Stage Renal Disease. Sci. World J..

[86] Langford C.P.H., Bowsher J., Maloney J.P., Lillis P.P. (1997). Social Support: A Conceptual Analysis. J. Adv. Nurs..

[87] Wang T., Molassiotis A., Chung B.P.M., Tan J.-Y. (2018). Unmet Care Needs of Advanced Cancer Patients and Their Informal Caregivers: A Systematic Review. BMC Palliat. Care.

[88] Gardiner C., Ingleton C., Gott M., Ryan T. (2011). Exploring the Transition from Curative Care to Palliative Care: A Systematic Review of the Literature. BMJ Support. Palliat. Care.

[89] Teike Lüthi F., Bernard M., Vanderlinden K., Ballabeni P., Gamondi C., Ramelet A.-S., Borasio G.D. (2021). Measurement Properties of ID-PALL, A New Instrument for the Identification of Patients with General and Specialized Palliative Care Needs. J. Pain. Symptom Manag..

[90] Müller E., Müller M.J., Boehlke C., Schäfer H., Quante M., Becker G. (2024). Screening for Palliative Care Need in Oncology: Validation of Patient-Reported Outcome Measures. J. Pain. Symptom Manag..

