# Supplementary material for: Evaluating Palliative Care Needs in Patients with Advanced Non-Malignant Chronic Conditions: An Umbrella Review of Needs Assessment Tools
Source: Healthcare (Basel). 2025 Dec 24;14(1):46. doi: 10.3390/healthcare14010046 (PMC12785391; doi:10.3390/healthcare14010046)
Supplement: Supplementary file 1 [file healthcare-14-00046-s001.zip › healthcare-4021629-supplementary.pdf]

Supplementary Files of Umbrella Review

Table S1. Reporting of PRIOR checklist items in this umbrella review.

PRIOR Item	Description	Where Reported in the Manuscript
1. Title	Identification as an umbrella review	Title / Abstract
2. Abstract	Structured summary including background, objectives, methods, results, conclusions	Abstract
3. Rationale	Justification for conducting the umbrella review	Introduction (paras 1–4)
4. Objectives	Clear statement of review objectives / questions	Introduction (last paragraph) & Methods – Design
5. Protocol and Registration	Registration in PROSPERO	Methods – Design (CRD42024553053)
6. Eligibility Criteria	PICOTSS-based inclusion/exclusion criteria	Methods – Eligibility Criteria & Table 1
7. Information Sources	Databases searched and coverage dates	Methods – Search Strategy
8. Search Strategy	Full electronic search strategies	Table 2 & Supplementary Tables S2–S4
9. Selection Process	Screening, reviewers, process description	Methods – Study Selection & Figure 1 (PRISMA)
10. Data Collection Process	Extraction methods and verification	Methods – Data Extraction
11. Data Items	All variables and outcomes extracted	Methods – Data Extraction & Table 4
12. Risk of Bias Assessment	Use of JBI tool for systematic reviews	Methods – Quality Appraisal & Table S5
13. Synthesis Methods	Narrative synthesis process; matrix method	Methods – Data Synthesis
14. Results of Included Reviews	Characteristics, aims, and findings	Results – Sections 3.1–3.5 & Tables 3–5
15. Discussion	Interpretation, implications, strengths & limitations	Discussion; Strengths and Limitations
16. Funding & Conflicts of Interest	Funding, acknowledgments, conflict statements	Acknowledgments / Funding / Conflict of Interest

Table S2. Search strategy Database: EBSCO host, CINAHL from inception to 30 June 2025.

Search ID	Search term	Result
+ Limiters (Full texts, published in the last 10 years, Human; Age Groups: All Adult; Language: English)	S13 AND S14 AND S15	56
S16	S13 AND S14 AND S15	586
S15	S10 OR S11 OR S12	230,676
S14	S5 OR S6 OR S7 OR S8 OR S9	443,353

Search ID	Search term	Result
S13	S1 OR S2 OR S3 OR S4	76,177
S12	(MH "Systematic Review") OR "systematic review"	190,039
S11	(MH "Meta Synthesis") OR "meta synthesis"	2,687
S10	(MH "Meta Analysis") OR "Meta-Analysis"	113,958
S9	"Screening tools"	4,157
S8	"Identification tool"	109
S7	"Diagnostic tool"	5,841
S6	(MH "Clinical Assessment Tools") OR "Assessment Tools"	203,116
S5	(MH "Needs Assessment") OR "Needs"	239,238
S4	(MH "Advance Care Planning") OR "Advance Care Planning	6,242
S3	(MH "Terminal Care") OR "Terminal care"	26,886
S2	(MH "Hospice Care") OR "Hospice care"	24,969
S1	(MH "Palliative Care") OR "palliative care"	52,481

Table S3. Search strategy Database: PubMed from inception to 30 June 2025.

	Search ID	Search term	Results
Concept Group A	1	"Palliative care" [Mesh]	6,379
	2	"Hospice and Palliative Care Nursing"[Mesh]	249
	2	"Terminal Care" [Mesh]	5,748
	3	"Advance Care Planning" [Mesh]	1,135
	4	"Hospice Care" [Mesh]	813
	5	"Advance Care Planning" [Text Word]	647
	6	"Hospice Care" [Text Word]	1,047
	7	"Hospice and Palliative Care Nursing" [Text Word]	255
	8	"Palliative Supportive Care" [Text Word]	9
	9	"Palliative Therapy "[Text Word]	219
	10	"Palliative Treatment" [Text Word]	771
	11	"Palliative Treatment*" [Text Word]	834
	12	"Palliative Care" [Text Word]	8,009
	13	"Terminal Care" [Text Word]	3,330
	14	"Palliative care" [mesh] OR "Hospice and Palliative Care Nursing" [mesh] OR "Terminal Care" [mesh] OR "Advance Care Planning" [mesh] OR "Hospice Care" [mesh] OR "Advance Care Planning" [Text Word] OR "Hospice Care" [Text Word] OR "Hospice and Palliative Care Nursing" [Text Word] OR "Palliative Supportive Care" [Text Word] OR "Palliative Therapy "[Text Word] OR "Palliative Treatment" [Text Word] OR "Palliative Treatment*" [Text Word] OR "Palliative Care" [Text Word] OR "Terminal Care" [Text Word]	13,574
Concept Group B	15	"Needs Assessment" [Mesh]	3,253
	16	"Assessment Tools" [Text Word]	1,440
		"Diagnostic tool*" [Text Word]	5,359
	17	"Identification tool" [Text Word]	62
		"Instruments" [Text Word]	
		"Screening tools" [Text Word]	900
	18	"Needs Assessment" [Mesh] OR"Assessment Tools"[Text Word] OR "Diagnostic tool*" [Text Word] OR "Identification tool"[Text Word] OR "Instruments" [Text Word] OR "Screening tools" [Text Word]	957,481
Concept Group C	19	"meta analysis" [MeSH Terms]	2,864
	20	"meta synthesis" [MeSH Terms]	793
	21	"meta analys*"[Text Word]	31,828
	22	"meta synthes*"[Text Word]	174
	23	"systematic review*" [Text Word]	34,502
	24	"review*" [Text Word]	452,248
	25	"Meta-Analysis" [MeSH Terms] OR "meta synthesis" [MeSH Terms] OR "meta analys*" [Text Word] OR "meta synthes*" [Text Word] OR "systematic review*" [Text Word] OR "review*" [Text Word]"	461,058
Concept Group A AND	35	("palliative care"[MeSH Terms] OR "Hospice and Palliative Care Nursing"[MeSH Terms] OR "Terminal Care"[MeSH Terms] OR "Advance Care Planning"[MeSH Terms] OR "Hospice	852

	Search ID	Search term	Results
Concept Group B AND *Concept Group C*		Care"[MeSH Terms] OR "Advance Care Planning"[Text Word] OR "Hospice Care"[Text Word] OR "Hospice and Palliative Care Nursing"[Text Word] OR "Palliative Supportive Care"[Text Word] OR "Palliative Therapy"[Text Word] OR "Palliative Treatment"[Text Word] OR "palliative treatment*"[Text Word] OR "palliative care"[Text Word] OR "Terminal Care"[Text Word]) AND ("Needs Assessment"[MeSH Terms] OR "Assessment Tools"[Text Word] OR "diagnostic tool*"[Text Word] OR "Identification tool"[Text Word] OR "Instruments"[Text Word] OR "Screening tools"[Text Word]) AND ("meta analysis as topic"[MeSH Terms] OR (("meta"[Journal] OR "meta"[All Fields]) OR "meta analys*"[Text Word] OR "meta synthes*"[Text Word] OR "sytematic review*"[Text Word] OR "review*"[Text Word])	
Concept Group A AND *Concept Group B* AND *Concept Group C* + Filters	36		23

Filters: Systematic Reviews, Meta-Analysis, Full texts, published in the last 10 years, Humans, English, Greek Modern, Adult: 19-44 years, Middle Aged + Aged: 45+ years, Middle Aged: 45-64 years, Aged: 65+ years, 80 and over: 80+ years.19+ years ➔ 23 Items

Table S4. Search strategy Database: PsycINFO from inception to 30 June 2025.

Search ID	Search term	Result
+ Limiters (*Linked Full Text; Publication Year: 2013-2023; Language: English; Age Groups: Adulthood (18 yrs & older); Population Group: Human*)	S7 AND S12 AND S16	9
S17	S7 AND S12 AND S16	44
S16	S13 OR S14 OR S15	89,113
S15	systematic review	58,891
S14	meta-synthesis	1,037
S13	meta-analysis	50,806
S12	S8 OR S9 OR S10 OR S11	36,517
S11	screening tools	10,186
S10	Diagnostic tool	5,132
S9	needs assessment tool	779
S8	needs assessment	21,934
S7	S1 OR S2 OR S3 OR S4 OR S5 OR S6	42,673
S6	hospice and palliative care	8,417
S5	end of life care	17,991
S4	terminal care	8,201
S3	hospice care	24,775
S2	advance care planning	2,020

Search ID	Search term	Result
S1	palliative care	22,741

Table S5. Risk of Bias Assessment of Included Systematic Reviews.

**Review (*n* =7)**												
**Authors**	**1**	**2**	**3**	**4**	**5**	**6**	**7**	**8**	**9**	**10**	**11**	**Comments**
1. Maas et al. (2013)	Y	Y	Y	Y	Y	Y	U	Y	NA	Y	Y	Methods used to minimize errors in data extraction not sufficiently detailed. No assessment of publication bias reported.
2. Stow et al. (2019)	Y	Y	Y	Y	Y	Y	Y	Y	Y	Y	Y	
3. ElMokhallalati et al. (2020)	Y	Y	Y	Y	Y	Y	Y	Y	Y	Y	Y	
4. Remawi et al. (2020)	Y	Y	Y	Y	Y	Y	Y	Y	Y	Y	Y	
5. Kirkland et al. (2022)	Y	Y	Y	Y	Y	Y	Y	Y	Y	Y	Y	
6. Kawashima & Evans (2023)	Y	Y	Y	Y	Y	Y	Y	Y	Y	Y	Y	
7. Xiu et al. (2023)	Y	Y	Y	Y	Y	Y	Y	Y	Y	Y	U	
Y=Yes, N=No, U=Unclear, NA= Not applicable												

1. Is the review question or aim explicitly stated?
2. Were the inclusion criteria appropriate for the review question?
3. Was the search strategy appropriate?
4. Were the sources and resources used to search for studies adequate?
5. Were the criteria for appraising studies appropriate?
6. Was critical appraisal conducted by two or more reviewers independently?
7. Were there methods to minimize errors in data extraction?
8. Were the methods used to combine studies appropriate?
9. Was the likelihood of publication bias assessed?
10. Were recommendations for policy and/or practice supported by the reported data?
11. Were the specific directives for new research appropriate?

Table S6. Potential Appraisal Tools Considered for Future Umbrella Reviews (COSMIN RoB & QUADAS-2).

Appraisal Tool	Purpose	Intended Application	Notes
**COSMIN Risk of Bias checklist**	Evaluate methodological quality of studies reporting psychometric properties (e.g., validity, reliability)	Future umbrella reviews including primary psychometric studies	Not applied in the current review
**QUADAS-2**	Evaluate risk of bias and applicability of diagnostic accuracy studies	Future inclusion of diagnostic accuracy studies in umbrella reviews	Not applied in the current review
